# Enzymatic Oxidation of Ca-Lignosulfonate and Kraft Lignin in Different Lignin-Laccase-Mediator-Systems and MDF Production

**DOI:** 10.3389/fbioe.2021.788622

**Published:** 2022-01-28

**Authors:** Markus Euring, Kolja Ostendorf, Martin Rühl, Ursula Kües

**Affiliations:** ^1^ Department of Molecular Wood Biotechnology and Technical Mycology, Büsgen-Institute, Georg-August-University of Göttingen, Göttingen, Germany; ^2^ Department of Wood Technology and Wood-based Composites, Burckhardt-Institute, Georg-August-University of Göttingen, Göttingen, Germany; ^3^ Department of Biology and Chemistry, Institute of Food Chemistry and Food Biotechnology, Justus Liebig University Giessen, Gießen, Germany; ^4^ Current address, Department of Food and Feed Improvement Agents, Fraunhofer Institute for Molecular Biology and Applied Ecology IME, Gießen, Germany; ^5^ Center for Molecular Biosciences (GZMB), Göttingen, Germany; ^6^ Center of Sustainable Land Use, University of Göttingen, Göttingen, Germany

**Keywords:** Ca-lignosulfonate, kraft lignin, basi-laccase *Cc*Lcc5, asco-laccase *Mt*L, mediator, guaiacol, 2,6-dimethoxyphenol, MDF

## Abstract

Laccase-mediator-oxidized lignin offers replacement for conventional chemical binders to produce fiberboards. Compared to the previously reported laccase–mediator system (LMS), a lignin-laccase-mediator-system (LLMS) has an advantage in that it requires much shorter fiber-enzyme incubation time due to significantly increased redox reactions. However, the cost of regularly applying laccase on an industrial scale is currently too high. We have employed *Cc*Lcc5 from cultures of the basidiomycete *Coprinopsis cinerea* as a novel basi-laccase (a CAZy subfamily AA1_1 laccase) in medium-density fiberboard (MDF) production, in comparison to the commercial formulation Novozym 51003 with recombinantly produced asco-laccase *Mt*L (a CAZy subfamily AA1_3 laccase-like multicopper oxidase from the ascomycete *Myceliophthora thermophila*). With the best-performing natural mediator 2,6-dimethoxyphenol (DMP), unpurified *Cc*Lcc5 was almost as good as formulated Novozym 51003 in increasing the molecular weight (MW) of the technical lignins tested, the hydrophilic high-MW Ca-lignosulfonate and the hydrophobic low-MW kraft lignin (Indulin AT). Oxygen consumption rates of the two distantly related, poorly conserved enzymes (31% sequence identity) with different mediators and lignosulfonate were also comparable, but Indulin AT significantly reduced the oxidative activity of Novozym 51003 unlike *Cc*Lcc5, regardless of the mediator used, either DMP or guaiacol. Oxygen uptake by both laccases was much faster with both technical lignins with DMP than with guaiacol. In case of lignosulfonate and DMP, 20–30 min of incubation was sufficient for full oxygen consumption, which fits in well in time with the usual binder application steps in industrial MDF production processes. LLMS-bonded MDF was thus produced on a pilot-plant scale with either crude *Cc*Lcc5 or Novozym 51003 at reduced enzyme levels of 5 kU/kg absolutely dry wood fiber with lignosulfonate and mediator DMP. Boards produced with *Cc*Lcc5 were comparably good as those made with Novozym 51003. Boards reached nearly standard specifications in internal bond strength (IB) and modulus of rupture (MOR), while thickness swelling (TS) was less good based on the hydrophilic character of lignosulfonate. LLMS-bonded MDF with Indulin AT and DMP performed better in TS but showed reduced IB and MOR values.

## Introduction

After cellulose, lignin ranks second as the most abundant renewable organic macromolecule on Earth. Lignin is one of the three main components of lignocellulosic biomass. It is considered as a pressure-tight amorphous matrix that reinforces and protects as a physical barrier the cellulose microfibril structure of lignified cell walls in vascular plants (Melro et al., 2021). In pulp and paper production, lignin is however an impurity that has to be removed from wooden fiber cells in various steps in order to obtain a pure cellulosic white paper (Smook, 2002). With a worldwide annual production of ∼70 Mio t in the pulping liquor, lignin is thus a technical mass by-product from the pulping industry, for which there is hardly any meaningful commercial use (Vishtal and Kraslawski, 2011; Wenger et al., 2020; Melro et al., 2021). Such technical lignins differ from the lignin in its native form. Their structure depends on the source of lignocellulose, be it from softwood, hardwood, or monocots with their different proportions of *p*-coumaryl alcohol, coniferyl alcohol, and sinapyl alcohol subunits in the protolignins, and above all on the method of extraction of a technical lignin. The largest amount of technical lignins (∼85%) results from alkaline kraft pulping with “white liquor” of NaOH and Na_2_S mixed with hot water (155–175°C), i.e., hydrophobic kraft lignin with an increased content of methoxyl groups and β-*O*-4-bonds and 2%–3% organically bound sulfur. It is present in the “black liquor” generated during fiber pulping through dissolution of lignin via ionization of phenolic groups, cleavage of β-aryl ether linkages, and condensation reactions. Kraft lignin is recovered from the “black liquor” by acid precipitation, which protonates phenols in the lignin under conformational changes that reduce electrostatic repulsion and hydrophilicity of lignin molecules and end up in lignin flocculation. Another 10% of technical lignins, i.e., hydrophilic lignosulfonates, originate from sulfite processing with SO_2_ and alkali metal or alkaline earth hydroxides. In the acidic sulfite process, α-*O*-4 ether bonds in lignin are cleaved and the *α*-carbons sulfonated, which makes the lignin soluble in a wide range of pHs. In addition, condensation reactions can take place between a benzylic carbon and another electron-rich carbon atom. By adding lime, sulfonated technical lignin can be precipitated from the spent sulfite liquor in the form of Ca salts. Another 5% of technical lignins, the soda lignin, originate from soda pulping, which is mainly applied to non-wood biomass. The strong alkali concentration (13%–16%) and the heating to 140–170°C to break non-phenolic β-*O*-4 bonds and form phenols also impair the cellulose and lead to an overall inferior-quality pulp. Furthermore, sulfur-free technical lignins with low molecular weight (MW) and higher homogeneity are obtained from organosolv pulping of lignocellulosic biomass as by-products of the second generation of bioethanol production (Ekielski and Mishra, 2021; Melro et al., 2021).

So far, only about 2% of technical lignins, mainly lignosulfonates (1.8 Mio t/year), are purified for commercial use. The remaining lignin is combusted for energy purposes or not used at all (Mohan et al., 2006; Wenger et al., 2020; Melro et al., 2021). Lignosulfonates were so far mainly used as additives in concrete in the building sector. They provide greater plasticity to mortar and control porosity, while the strength, diffusivity, and hydraulic conductivity of the cement remain (Klapiszewski et al., 2019; Melro et al., 2021). Small volume and niche applications are verified in usage of nontoxic technical lignins, e.g., in feed pellet production and as UV absorber, as activated carbon, and as replacement for carbon black (Puls, 2009; Higson and Smith, 2021). Although little has yet been implicated on an industrial scale, research on lignins revealed a huge potential for valorization in applications to serve as fillers, reinforcing agents, adhesives, emulsifiers, and dispersants, as well as feedstock of low cost and specialty chemicals, gels, nanoparticles, absorbers, flocculants, composites, biofoams, bioplastics, sensors and electrodes, and other novel biomaterials (Upton and Kask, 2015; Kai et al., 2016; Collins et al., 2019; Yang et al., 2019; Zhang et al., 2019; Jiang et al., 2020; Moreno and Sipponen, 2020; Wang et al., 2020; Yu and Kim, 2020; Aristri et al., 2021; Karthäuser et al., 2021; Kumar et al., 2021; Liu et al., 2021; Ma et al., 2021; Melro et al., 2021).

Because they are available in large quantities, lignosulfonates and kraft lignin are most interesting polyphenols for the development of binders for wood composites. Due to the high content of phenolic groups, both technical lignins are considered to be very suitable green substitutes in existing binder systems (Mansouri et al., 2007; Tejado et al., 2007; Norström et al., 2018; Jiang et al., 2020; Wang et al., 2020; Aristri et al., 2021; Karthäuser et al., 2021; Kumar et al., 2021; Ostendorf et al., 2021). Nonetheless, extensive research is required before a successful industrial implication. Blends of conventional phenol-formaldehyde (PF) resins with lignosulfonate (from 10:5 to 6:5) added to 2:1 beech:cerris fibers from thermomechanical pulping (TMP), for instance, increased water absorption and thickness swelling (TS) and decreased bending strength of medium-density fiberboard (MDF) pressed at 190°C. The hydrophilic lignosulfonate however acted in the system only as a filler because lignin does not hydrolyze at this pressing temperature (Yotov et al., 2015). Adding hydrophobizers could be a solution to improve moisture resistance, but this may hinder good gluing (Mathiasson and Kubát, 1995; Kües et al., 2007; Kirsch et al., 2016). Use of a novel hot-air/hot-steam curing to achieve higher temperatures for lignin repolymerization under reduction of press times (Euring et al., 2015; Euring et al., 2016) improved physical properties of panels produced with lignin admixture (Kirsch et al., 2018). Modifying lignin prior to admixture in resins can make them more efficient as functional eco-friendly glues (Garajová et al., 2021; Karthäuser et al., 2021). The first blends of binder with technical lignin are now on the market (https://www.wisaplywood.com/wisa-biobond/; accessed August 18, 2021).

One possible kind of lignin activation prior to use is enzymatic (Suurnäkki et al., 2010; Taboada-Puig et al., 2013; Munk et al., 2018; Perna et al., 2019). Laccases (EC 1.10.3.2; *p*-diphenol oxygen oxidoreductases) are multi-copper-oxidases for bound copper-atoms and belong to the most important lignin depolymerizing and polymerizing enzymes in nature (Mayer and Staples, 2002; Hoegger et al., 2006; Rodríguez Couto and Toca Herrera, 2006; Kües and Rühl, 2011; Kües, 2015; Munk et al., 2015; Munk et al., 2017). Laccases are produced by many fungi (especially many by white-rot basidiomycetes), plants, bacteria, and insects. They act with low specificity and variable efficiency on different *o*- and *p*-diphenols as well as aromatic amines, under reduction of four electrons from molecular oxygen to water (Mayer and Staples, 2002; Hoegger et al., 2006; Kües and Rühl, 2011; Kües, 2015). Due to their broad substrate range, laccases are attractive for many biotechnological applications. This includes processes involving lignin, for instance, to break down the unwanted lignin in “biobleaching” in the paper and textile industries, to optimize unhindered access and the transformation of all polymers in lignified cell walls in biorefinery, to functionalize lignin by grafting, and to modify lignified cell walls for fiber auto-adhesion in fiberboard production for better green bonding (Hüttermann et al., 2001; Mayer and Staples, 2002; Mai et al., 2004; Kloeser et al., 2007; Munk et al., 2017a; Kunamneni et al., 2008; Widsten and Kandelbauer, 2008; Kües, 2015; Zerva et al., 2019).

Commercial fiberboards are obtained from hot-pressing wood fibers mainly from TMP with the addition of suitable petrochemical glues (Dunky and Niemz, 2002; Müller et al., 2007). There is considerable interest to replace conventional adhesives in MDF production by sustainable green alternatives, including enzymatic solutions (Kües et al., 2007; Kirsch et al., 2016; Perna et al., 2019; Ostendorf et al., 2020). TMP fibers have a plasticized glassy lignin layer on the surface, due to heating above the glass transition point of lignin during the TMP process (Mai et al., 2004; Kloeser et al., 2007; Kües et al., 2007; Widsten and Kandelbauer, 2008). This lignin on the fiber surfaces can become activated through oxidation processes and radicalization of phenolic lignin groups with laccases (Felby et al., 1997; Kharazipour et al., 1997; Unbehaun et al., 2000; Felby et al., 2002; Widsten and Kandelbauer, 2008a; Euring et al., 2011). During hot-pressing to MDF, these phenoxy radicals help in coupling or substitution reactions with other molecules to create in-fiber self-bonding stable connections between wood fibers (Unbehaun et al., 2000; Felby et al., 2002; Mai et al., 2004; Kües et al., 2007; Widsten and Kandelbauer, 2008). However, the incubation times of an effective enzymatic activation of TMP fibers for auto-adhesion were too long for a cost-effective industrial application (Garajová et al., 2021). This shortcoming in MDF production can be remedied with a laccase–mediator system (LMS) (Euring et al., 2011; Euring et al., 2013; Garajová et al., 2021). Mediators are small redox molecules, which are oxidized by laccase to radicals in cycles to react as electron carriers with other organic phenolic and non-phenolic compounds to oxidize these. In LMS, therefore, with the recyclable mediators, a laccase is indirectly able to activate more phenolic and also non-phenolic compounds on the TMP fibers (Bourbonnais and Paice, 1990; Kües, 2015). Incubation times of wood fibers for activation and auto-adhesion were drastically reduced with LMS from several hours to a few minutes, which corresponds to the usual gluing time in commercial MDF production. Moreover, LMS-MDF produced on a pilot scale offered good physical technological properties in bending and internal bond strength (IB) and in TS (Euring et al., 2011; Euring et al., 2013). For further implementation, the efficiency of LMS towards lignin modification should be further optimized. Up to now, a commercial laccase preparation of a brown liquid designed for applications of paper pulp delignification [Novozym 51003 (Novozymes, 2007)] has been used in LMS to make MDF in a pilot plant scale (Euring et al., 2011; Euring et al., 2013). Novozym 51003 contains a thermostable laccase recombinantly produced from a gene of the ascomycete *Myceliophthora thermophila* (*Mt*L) cloned in *Aspergillus oryzae* and is applicable for the oxidation of a broad range of lignocellulosic materials (Novozymes, 2007; Lund et al., 2016; Ernst et al., 2018). Earlier LMS studies required a 1 L incubation solution of 100 U/ml of this asco-laccase (Euring et al., 2011; Euring et al., 2013; Euring et al., 2016) or even 200 U/ml (Kirsch et al., 2016), per 1 kg fiber mass (atro, for “absolut trocken,” absolutely dry) for the production of MDF of good technical quality at the pilot plant scale. Due to its price [around 30€ per liter in year 2020 (Univar, 2020)], it could still be difficult to integrate the LMS into a cost-oriented MDF company.

The first approaches to produce fiberboards were made by applying technical lignins in conjunction with a laccase as a “two-component-system,” resulting in lignin activation and enhanced self-bonding of wood fibers (Hüttermann et al., 2001; Mai et al., 2004). Incorporation of lignin into the LMS (aka LLMS) (Euring et al., 2016a) and the search for alternative mediators (Kirsch et al., 2015; Kirsch et al., 2016) and alternative laccases that work well within the LMS (Gouveia et al., 2018; Braunschmid et al., 2021; Garajová et al., 2021) are approaches for further process optimization and potential cost reduction. From an economic point of view, a first MDF pilot plant study in LLMS with lignosulfonate and either caffeic acid or vanillyl alcohol as a mediator indicated a good possibility to set the laccase activity lower with still consistently good technical product values as in the work before that used 100 kU Novozym 51003/kg of atro wood fiber and 10 mM of a mediator (Euring et al., 2016a).

In this study, we used a 200-fold-lower enzyme amount (5 kU/kg atro wood fiber in a diluted concentration of 5 U/ml) as before in LLMS (Euring et al., 2016a) and compared the asco-laccase *Mt*L in Novozym 51003 (Novozymes, 2007) with the distantly related enzyme *Cc*Lcc5 as the best-expressed basi-laccase from the laccase multi-gene family of the basidiomycete *Coprinopsis cinerea* (Kilaru et al., 2006; Rühl et al., 2013). We applied either Novozym 51003 or *Cc*Lcc5 in purified or unpurified form, combined with 2,6-dimethoxyphenol (DMP) and guaiacol (2-methoxyphenol) as natural mediators in LLMS with pine fibers and either Ca-lignosulfonate or kraft lignin (Indulin AT), respectively. Potentials of laccases to oxidize lignin were analyzed by oxygen consumption tests and in gel-permeation chromatography (GPC) to determine MW changes in the Ca-lignosulfonate and the kraft lignin. MDF with a thickness of 8 mm and a density of 780 kg/m^3^ was manufactured in LMS and LLMS.

## Materials and Methods

### Materials

Ca-lignosulfonate was produced by sulfite processing from Scandinavian softwood (spruce) and provided under the brand name Borrement Ca 120 [Zig] by Borregaard LignoTech (Karlsruhe, Germany). Indulin AT (kraft lignin) was produced by MeadWestvaco Corporation (Richmond, Virginia, United States) from pine and precipitated from black liquor of liner-board-grade pulp (Hu et al., 2016). DMP with a chemical purity of 99% and guaiacol with a chemical purity of 98% were purchased from Alfa Aesar (Karlsruhe, Germany). Commercial *M. thermophila* enzyme Novozym 51003 (Novozymes, 2007) recombinantly produced by *A. oryzae* was received from Novozymes (Bagsvaerd, Denmark). The activity of the laccase stock was about 1,000 U/ml, confirmed with 2,2′-azino-di-(3-ethylbenzothiazoline-6-sulfonic acid) (ABTS) (AppliChem, Darmstadt, Germany) according to Matsumura et al. (1986). *C. cinerea* Lcc5 (*Cc*Lcc5) basi-laccase was produced by cultivating an *lcc5* overexpression transformant [*C. cinerea* FA2222 pYSK20 clone 11 (Kilaru et al., 2006)] in YMG medium (per liter: 4 g yeast extract, 10 g malt extract, and 4 g glucose; Rao and Niederpruem, 1969). All further chemicals, solvents, eluents, etc. for the *Cc*Lcc5 production and purification, enzymatic reactions, and GPC were ordered either from AppliChem (Darmstadt, Germany), Alfa Aesar (Karlsruhe, Germany), or Sigma-Aldrich (Seelze, Germany). Wood fibers for MDF production were from Steico SE (Czarnkow, Poland) and produced via TMP from a mixture of *Pinus sylvestris* and *Pinus radiata* wood (80% + 20%). Industrial urea-formaldehyde (UF) resin (UK K350; 68% solids) was from BASF (Ludwigshafen, Germany). Hydrophobing agent HydroWax 138 (a paraffin emulsion, 60% solids) was purchased from Sasol Wax GmbH (Hamburg, Germany). McIlvaine buffer was composed of 0.2 M K_2_HPO_4_ and 0.1 M citric acid, buffered to pH 6.0, as the optimal pH determined before for reactions of Novozym 51003 with both natural and technical lignins (Euring, 2008).

### Basi-Laccase *Cc*Lcc5 Production and Purification

Three YMG agar blocks (1 × 1 cm) of freshly grown mycelium of FA2222 pYSK20 clone 11 were used for inoculation of 50 ml liquid YMG pre-cultures in 500 ml flasks. After 4 days of incubation at 37°C as standing cultures, pre-cultures were homogenized by an ULTRA-TURRAX (IKA, Staufen, Germany) for 30 s at 8,000 rpm and for 30 s at 9,500 rpm. Each 5 ml of suspension was used for the main cultures in 500 L flasks with 100 ml YMG medium containing 0.1 mM CuSO_4_. Main cultures were incubated at 37°C on a shaker (Infors HT Multitron Standard, Infors GmbH, Einsbach, Germany) at 120 rpm. Every day, samples were taken, and laccase activity was measured spectroscopically with 0.5 mM ABTS at 420 nm (*ε* = 36,000 M^−1^ cm^−1^) in 100 mM Na acetate buffer, pH 5.0, following Matsumura et al. (1986), until the activity was around 5.5 U/ml (one unit of activity was defined as the amount of enzyme needed to oxidize 1 µmol of the substrate per minute), and cultures were harvested. After filtration of the fungal cultures through Whatman No. 1 paper (Whatman, Dassel, Germany) with a suction filter, supernatants for analyses with unpurified basi-laccase (hereinafter referred to as *Cc*Lcc5 in comparison to the purified *Cc*Lcc5) were stored at 4°C until further use (see sections below). To purify, 4.35 L of fresh culture supernatant (pH 7.2) was adjusted to pH 6.4 and then centrifuged at 4°C with 10,000 *g* for 30 min (Beckman J2-HS, Beckman Coulter, Krefeld, Germany) to separate the laccase-containing supernatant from any mycelial fragments left. Next, cross-flow filtration of the supernatant with a PALL filter cassette of 1 MDa (OMEGA membrane, Pall Corporation, Dreieich, Germany) was performed. This was followed by chromatographic steps with an ÄKTA-FPLC system (GE Healthcare, Freiburg, Germany) at pH 6.4, using two anion-exchanging columns (DEAE Sepharose Fast Flow, GE Healthcare Biosciences, Uppsala, Sweden) with a loading buffer of 20 mM KH_2_PO_4_ and an elution buffer of 20 mM KH_2_PO_4_, 1 M NaCl. The last step of purification took place by hydrophobic interaction chromatography (HIC) with a Phenyl Sepharose Fast Flow Column [loading buffer 20 mM KH_2_PO_4_ + 1 M (NH_4_)_2_SO_4_ and elution buffer 20 mM KH_2_PO_4_]. After purification, laccase activity was determined as being above 236 U/ml.

### Laccase Sequences

Sequences of asco-laccase *Mt*L (GenBank accession number AEO58496) and basi-laccase *Cc*Lcc5 (DAA04510) were aligned with each other using the program ClustalX2 (Larkin et al., 2007). Sequence identity values were determined with the pBlast tool at NCBI (https://www.ncbi.nlm.nih.gov/). Domain structures of *Mt*L were assigned according to the established enzyme structure, and amino acids (aa) important for function were marked (Kilaru et al., 2006; Ernst et al., 2018).

### Lignin-Laccase Incubations and MW Determination by GPC

GPC is a method to determine the MW distribution of phenolic substances like lignin. Once lignin or another phenolic compound is oxidized by a laccase, changes in the MWs by further reactions can be recognized (Euring et al., 2016a). A high-pressure liquid chromatography (HPLC) unit from Waters (Milford, Massachusetts, United States) was used, containing a Waters e2695 Separation Module and a Waters 2489 UV detector. Lignin prior or after reactions was separated by size through a series of columns (from Showa Denko K.K., Tokyo, Japan), first a Shodex KF-G column (maximum pore size of 8 µM) to remove any impurities, then a Shodex KF-803 (maximum pore size of 500 µM), and a Shodex KF-804 column (maximum pore size of 1,500 µM). Lignin was detected by the UV detector at 254 nm. Without an enzymatic reaction, lignosulfonate peaked in the chromatograms broader at 13.5–14.5 min, Indulin AT at 15.5–16.0 min, DMP at 20.8–21.0 min, and guaiacol at 20.0–20.5 min. After LLMS treatments, the peak of lignosulfonate moved closer to 14.5 min and Indulin closer to 16.0 min, indicating size increases, while peaks of mediators remained in the chromatograms on their relative positions.

For sample preparation for GPC, 0.5 g lignin (lignosulfonate or Indulin AT) was incubated for 15, 30, and 60 min in conical reaction flasks (250 ml) in 125 ml McIlvaine buffer (pH 6.0), containing 5 U/ml laccase (either Novozym 51003 or unpurified or purified *Cc*Lcc5 laccase) and 10 mM of either mediator DMP (lignin:mediator 1:0.26, w/w) or mediator guaiacol (lignin:mediator 1:0.32, w/w), respectively. Reference samples contained either no laccase or no mediator or no laccase and no mediator. Each of the different reactions was repeated three independent times. All reactions were performed at room temperature (RT) as the typical temperature used in industrial fiberboard production (Euring et al., 2011). The reaction flasks with the samples were closed airtight and placed for incubation onto a slow rotation shaker GFL 3015 (Gesellschaft für Labortechnik, Burgwedel, Germany). After incubation, the samples were briefly brought to a boil to stop the laccase actions. Per test sample, three aliquots of 200 µl were filled into individual vials and measured in GPC as three technical repeats. The eluent used was a water/acetonitrile solution (80/20, v/v) with a pH of 7.8. Previously measured polystyrene standards with MWs from 2,000 to 150,000 Da were used as reference values for data analysis by the Empower 3 Personal program (Waters Corporation, Milford, Massachusetts, United States). Mean values and standard deviations were calculated. Significant difference between the means was assessed with Tukey’s Honestly Significant Difference (HSD) test with a significance level of *p* < 0.05 using the software SPSS version 26 (IBM, Armonk, NY).

### Oxygen Consumption Measurements

Oxygen consumption measurements were carried out following the methods of Widsten (2002) and Grönqvist et al. (2005), modified by Kirsch et al. (2015), as a means of a quantitative analysis of the laccase activity in relation to its oxidation processes on phenolic substrates (here technical lignins or a mediator or both). Reactions took place in a room with a constant temperature of 23°C. McIlvaine buffer (pH 6.0), containing 10 mM of either DMP or guaiacol, was heated to 60°C with stirring for 30 min to allow the mediator to dissolve more easily. Then, the buffer was cooled down to RT. McIlvaine buffer (pH 6.0), with or without dissolved mediator, was ventilated for 0.5 h under stirring with compressed air to achieve 100% saturation with oxygen. For each test sample, 0.5 g lignin (lignosulfonate or Indulin AT) was added into a conical reaction flask of a total volume of 125 ml. Flasks were then completely filled to the brim with aerated McIlvaine buffer (pH 6.0), with or without a dissolved mediator (lignin:DMP 1:0.26, w/w; lignin:guaiacol 1:0.32, w/w), and sealed airtight with screw caps with rubber septa equipped with an O_2_ electrode InPro 6800 (Mettler Toledo, Urdorf, Switzerland) and an inlet and an outlet cannula. After a calibrating phase of the O_2_ electrode for about 5 min with stirring, the O_2_ content in the buffer was set at 100% saturation (8 ppm). Reactions were started by injecting 5 U of a laccase (either Novozym 51003 or unpurified or purified *Cc*Lcc5) into a flask through the inlet cannula, while excess liquid was immediately released from the outlet cannula. Reference samples without enzyme were run for all sample types with lignin. Reference samples with no lignin contained pure buffer either with or without a dissolved mediator, with or without enzyme. Every test situation was repeated three times. The O_2_ content was recorded in samples every 10 s by an O_2_ transmitter 4100e (Mettler Toledo, Urdorf, Switzerland) over an incubation time of 180 min. The O_2_ consumption was calculated relative to the O_2_ content with the program LabVIEW 7.1 (National Instruments, Austin, Texas, United States). Mean values were calculated with the LabVIEW 7.1 program and consumption curves drawn. Standard deviations were always below 1.2% (not further shown here).

### Pilot Plant Production of LLMS-Bonded MDF

For LLMS-bonded MDF production, 5 kU laccase was applied per kilogram of atro wood fiber to meet the technical requirements of MDF (DIN EN 622-5, 2010). Commercial Novozym 51003 with recombinant asco-laccase *Mt*L (1,000 U/ml) was diluted 200-fold using McIlvaine buffer (pH 6.0), and 15 L solution (end concentration 5 kU/l) was added to 15 kg of atro wood fiber. Basi-laccase *Cc*Lcc5 was used unpurified as obtained from fermentation (in total 13.39 L with 5.6 U laccase/ml), filled up with McIlvaine buffer (pH 6.0) to a volume of 15 L. The amount of mediator DMP was set to 10 mM per kilogram of atro wood fibers. Lignins (lignosulfonate or Indulin AT; particle sizes ≤10 µm) were added to formulations at 10% (w/w) atro wood fibers. Reference solutions with enzyme contained no lignin (as LMS), no mediator, or no lignin and mediator. Reference solutions without enzyme were pure McIlvaine buffer (pH 6.0), the buffer with mediator, with lignin, or with both. For enhanced hydrophobicity of produced panels, 1% of HydroWax 138 was directly mixed into all formulations. As a standard reference, UF K350 was used at a resin load of 10%, together with 1% of HydroWax 138 emulsion and 2% of ammonium sulfate solution as hardener (Bütün-Buschalsky and Mai, 2021). Fibers were treated with the formulations, based on Euring et al. (2016a), using an MDF pilot plant (Binos, Springe, Germany). In brief, for each panel variant, 15 kg of dried pine TMP fibers (atro) was transferred with a conveyer belt into a horizontal blender unit where they were sprayed with the respective formulations by means of high-pressure injectors with spray nozzles (1.5 mm in diameter). The moisture content of fibers rose thereby to 40%. Then, the fibers passed with an inlet temperature of 120°C through a tube dryer, where drying took place at 60–70°C to a final moisture content of 12%–15%. The 15 kg wood fibers were further transferred via a cyclone and another conveyor belt into the fiber bunker, which scattered them into a continuous homogenous fiber mat for six boards in total, with the target density of 780 kg/m^3^ each. Fiber fleece for panel dimensions of 630 mm in length, 450 mm in width, and 8 mm in thickness was pre-pressed at RT and hot-pressed to MDF at 200°C and an operating pressure of 200 bar. It took about 30 min from spraying the wood fibers to hot-pressing. Controlled by laccase activity tests (Matsumura et al., 1986), the enzymes in the material were 99% functional until after the pre-pressing when they were finally deactivated by the hot-pressing. Reference MDF made with UF resin was manufactured using the same procedure as described above, with the exception of drying in the cyclone at 60°C. The process of MDF production was repeated twice for each variant (*n* = 3 runs × 6 boards each). After a conditioning phase for at least 48 h at 20°C and 65% relative humidity (RH), panels were cut into test specimens according to selected test methods: IB (*n* = 6 per board) (DIN EN 319, 1993), modulus of rupture (MOR, *n* = 3 per board) (DIN EN 310, 1993), and TS (*n* = 6 per board) (DIN EN 317, 1993). Mean values and standard deviations were calculated. Significant difference between the means was assessed with Tukey’s HSD test with a significance level of *p* < 0.05 using the software SPSS version 26.

## Results and Discussion

### Sequence Comparison of the Asco-Laccase *Mt*L and the Basi-Laccase *Cc*Lcc5

**FIGURE 1:**
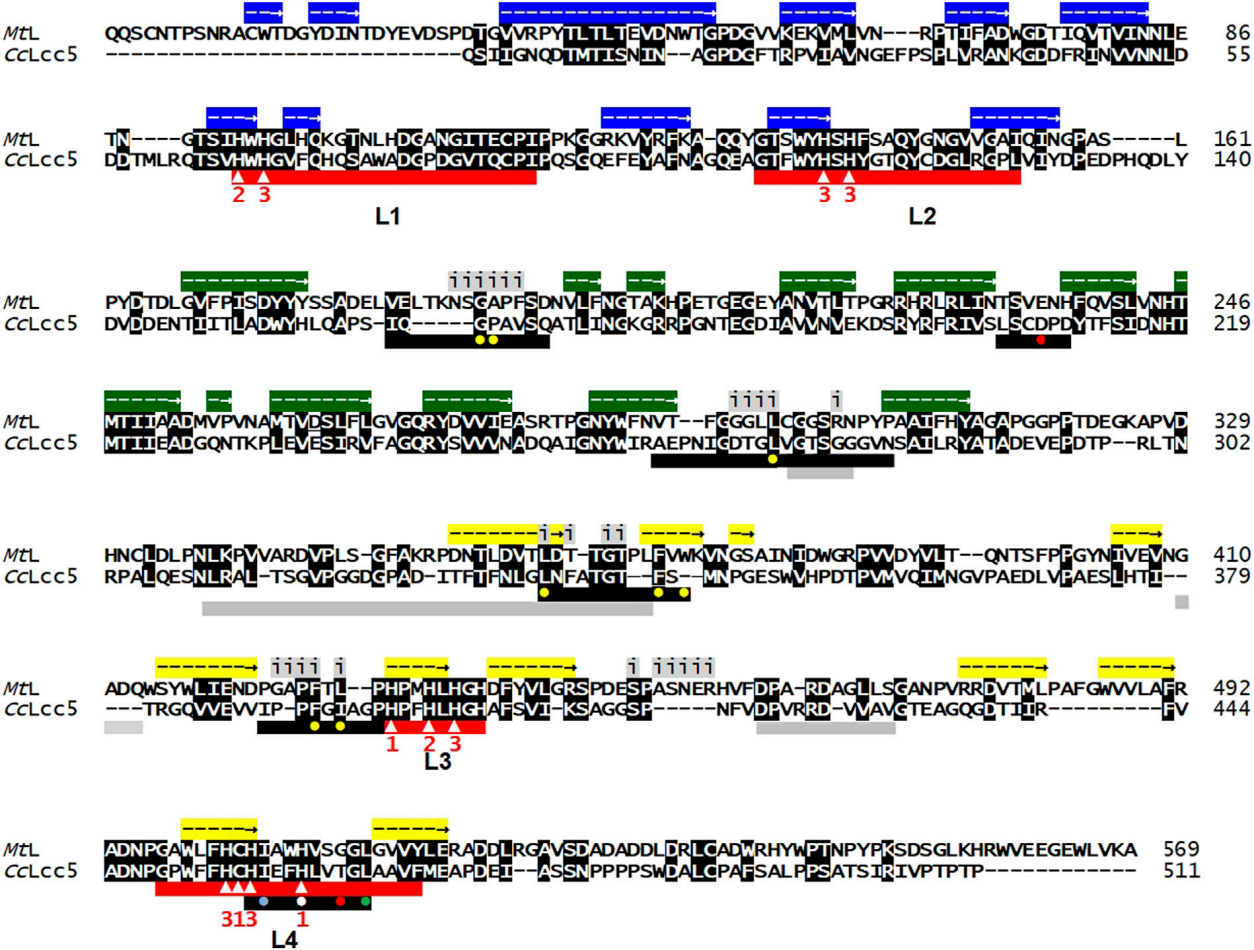
Sequence alignment of asco-laccase *Mt*L (AEO58496) and basi-laccase *Cc*Lcc5 (DAA04510). Conserved amino acids are shown with white letters in black boxes. The three cupredoxin-like domains are marked above the *Mt*L sequence with arrows in colored boxes for extension of their helical structures (blue; cupredoxin-like domain 1; green: cupredoxin-like domain 2; yellow: cupredoxin-like domain 3), according to Ernst et al. (2018). Residues located at the unique dimerization interface in the asco-laccase *Mt*L are marked above the alignment with letter i (for interface) underlain in grey (Ernst et al., 2018). Red bars beneath the alignments mark the conserved laccase signature sequences L1 to L4, with 10 conserved histidines and a conserved cysteine indicated by white triangles within the bars. Numbers 1, 2, and 3 beneath show the ligands for binding of T1, T2, or T3 copper. Black bars mark the loops forming the substrate pocket in the folded proteins (Hoegger et al., 2006; Kilaru et al., 2006; Kües and Rühl, 2011; Ernst et al., 2018). The hydrophobic residue Leu in L4, regarded as an indicator for enzymes with medium-redox-potential (Hoegger et al., 2006; Kües and Rühl, 2011), is marked in the black bar by a filled green circle. Yellow filled circles in black bars stand for hydrophobic residues that line the T1-Cu pocket of *Mt*L, the white and red filled circles for His508 and Glu235 for the polar recognition motif of *Mt*L for phenolic substrate (DMP) deduced in analogy from the established structure of DMP-bound *M. albomyces* laccase *Ma*L, and a light-blue filled circle for a further hydrophobic ligand in the sequence of motif L4 (after Ernst et al., 2018). Grey bars underneath the alignments show sequences for the proposed alternative binding site for non-phenolic ABTS on the surface of folded *Mt*L with an unusual hole localized 26 Å from the T1-Cu pocket (Ernst et al., 2018) in analogy to *Bacillus subtilis* CotA laccase (Liu et al., 2016).

Laccases consist of three cupredoxin-like domains and are characterized by the laccase signature sequences L1 to L4 with 10 conserved histidines and one conserved cysteine as binding sites for the four copper atoms. Mononuclear Type 1 Cu (T1) binds at motifs L3 and L4 in the cupredoxin-like domain 3, and mononuclear Type 2 Cu (T2) and binuclear Type 3 Cu (T3) at the trinuclear copper center provided in the three-dimensional folded enzymes by L1 to L4 at the interfaces between cupredoxin domains 1 and 3. Substrate-binding pockets are formed by variable sequences localized within the cupredoxin-like domain 2 and by residues at the T1-Cu pocket from L4 (Hoegger et al., 2006; Kües and Rühl, 2011; Ernst et al., 2018; Figure 1). Basi-laccase *Cc*Lcc5 of *C. cinerea* and asco-laccase *Mt*L of *M. thermophila* are both classified in the auxiliary activity (AA) family 1 of the CAZy database, but *Cc*Lcc5 falls into the distinct subfamily AA1-1 with typical basidiomycete *sensu stricto* laccases and *Mt*L into AA1-3 with ascomycete laccase-like multicopper oxidases (Kilaru et al., 2006; Levasseur et al., 2013; Ernst et al., 2018). The mature basi-laccase *Cc*Lcc5 comprises 511 aa, minus the N-terminal secretion signal (Kilaru et al., 2006; Figure 1). The recombinant mature asco-laccase *Mt*L has a length of 599 aa, by an extended N-terminus and a C-terminal plug as typical for AA1_3 enzymes (Ernst et al., 2018; Figure 1). The two enzymes are comparably poorly conserved in overall protein sequence (31% aa identity), with the highest identity in the first and the third cupredoxin-like domains (both 40% aa identity), followed by the second domain (36% aa identity). Both enzymes have a hydrophobic Leu in the L4 motif 10 residues downstream of the conserved Cys (Figure 1), which is considered to be typical for medium-redox-potential enzymes (Hoegger et al., 2006; Kües and Rühl, 2011). In line with this, the two enzymes have similar medium-redox-potentials measured as 0.47 and 0.54 V, respectively (Li et al., 1999; Rühl, 2010).

Interactions of laccases with their possible substrates are generally poorly understood. Structural analysis of *Mt*L with the phenolic DMP in analogy to asco-laccase *Ma*L of *Melanocarpus albomyces* indicated recognition by the polar His508 and Glu235 as well as several hydrophobic residues that line the T1-Cu pocket of *Mt*L (Ernst et al., 2018; Figure 1). Some of these residues are actually conserved also in the predicted substrate pocket of *Cc*Lcc5, which otherwise consists of loops of different lengths and variable sequence composition as compared to *Mt*L (Figure 1). The likely unique binding site for the larger artificial non-phenolic substrate ABTS on the outer surface of *Mt*L also differs from the sequences present at the corresponding place in *Cc*Lcc5 (Figure 1) and from all known structures of other basi-laccases (Liu et al., 2016; Ernst et al., 2018). In fact, laccase reactions are determined by specific interplays between the enzyme sequence and structure on the one hand and respective substrate properties on the other. The high variability in general and especially in substrate pockets in different laccase sequences and the large variety of possible substrates however make it impossible to rationally predict efficiencies in reactions of a laccase with a given substrate (Pardo and Camarero, 2015; Mehra et al., 2018), i.e., in this study whether basi-laccase *Cc*Lcc5 behaves in a comparable way to the distantly related asco-laccase *Mt*L with technical lignins and with the mediators chosen.

For further comparison, the well-studied asco-laccase *Mt*L has an optimal pH with syringaldazine as a phenolic substrate at pH 6.5 to 7.0 and with the non-phenolic ABTS at pH 2.7 (Berka et al., 1997; Xu, 1997) and the novel basi-laccase *Cc*Lcc5 at pH 7.0 and 5.0 (Rühl, 2010), indicating a good potential for enzymatic activities of *Cc*Lcc5 with substrates around the neutral pH. Indeed, pH values in the neutral range are best suited for laccase-catalyzed lignin polymerization reactions (Euring, 2008; Huber et al., 2016a; Ortner et al., 2018), among other things through a more open structure of lignosulfonates at pH 7.0 for better enzymatic access and for Indulin AT the restricted solubilization behavior at low pH (Huber et al., 2016a; Braunschmid et al., 2021; Mayr et al., 2021). These arguments together underline our investigation of the quality of basi-laccase *Cc*Lcc5 in experimental tests as an alternative to the asco-laccase *Mt*L as functional enzymes in LLMS for MDF production.

### Detection of MWs of Technical Lignins via GPC—Effects of Different Laccases and Mediators

Lignosulfonate and Indulin AT had an initial MW of 58 and 8 kDa, respectively. Incubation in McIlvaine buffer (pH 6.0) had no effect on the MWs, with no or only little addition of a mediator (Table 1). In contrast, as also shown in Table 1, most enzymatic treatments with either asco-laccase *Mt*L or basi-laccase *Cc*Lcc5, without or with addition of a mediator, increased significantly the average MW of the technical lignins, similar to that reported in our previous study with Novozym 51003 and lignosulfonate, and either caffeic acid or vanillyl alcohol as mediators (Euring et al., 2016a). The results suggest that both laccases oxidized, directly or indirectly, the lignins and that these secondarily reacted by polymerization via modified phenolic groups, in accordance with earlier observations on different laccases with lignins (Johansson et al., 2014; Ortner et al., 2015; Euring et al., 2016a; Gouveia et al., 2018; Braunschmid et al., 2021) and with mediators and lignins (Ortner et al., 2015; Euring et al., 2016a) where the presence of laccase-oxidized mediators resulted in an additional and enhanced lignin radicalization (Bourbonnais and Paice, 1990; Potthast et al., 1995; Potthast et al., 1999; Rittstieg et al., 2002; Kües et al., 2007; Areskogh et al., 2010; Prasetyo et al., 2010; Euring et al., 2011).

**TABLE 1:** Changes in MWs of technical lignins due to incubation with laccases and LMSs for different times in McIlvaine buffer (pH 6.0).

Technical lignin	Enzyme	Mediator	MWs in kDa after incubation^a^		
			15 min	30 min	60 min
Lignosulfonate	—	—	58.0 ± 2.2 (a, 1)	58.0 ± 2.0 (a, 1)	58.0 ± 3.0 (a, 1)
		DMP	59.7 ± 1.2 (a, 1)	60.1 ± 1.3 (a, 1)	60.5 ± 1.2 (a, 1)
		Guaiacol	58.6 ± 1.1 (a, 1)	59.1 ± 1.4 (a, 1)	60.1 ± 1.5 (a, 1)
	Purified *Cc*Lcc5	—	60.7 ± 1.8 (a, 1)	60.5 ± 0.5 (a, 1)	60.0 ± 0.5 (a, 1)
		DMP	62.9 ± 2.5 (b, 1)	67.9 ± 2.0 (b, 2)	65.0 ± 2.0 (b, 1)
		Guaiacol	62.0 ± 1.5 (a, 1)	63.2 ± 0.2 (b, 1)	62.0 ± 1.0 (b, 1)
	*Cc*Lcc5	—	62.7 ± 1.2 (b, 1)	65.5 ± 1.9 (b, 1)	66.0 ± 1.5 (b, 1)
		DMP	82.9 ± 1.6 (d, 1)	87.9 ± 2.6 (e, 2)	85.0 ± 1.5 (d, 2)
		Guaiacol	72.0 ± 0.5 (c, 1)	73.2 ± 2.5 (c, 1)	72.0 ± 2.0 (c, 1)
	Novozym 51003	—	67.0 ± 1.1 (b, 1)	69.5 ± 2.7 (b, 1)	65.2 ± 0.2 (b, 1)
		DMP	92.9 ± 2.6 (f, 1)	97.9 ± 1.8 (f, 2)	95.0 ± 1.4 (f, 2)
		Guaiacol	82.0 ± 2.0 (d, 1)	83.2 ± 1.2 (d, 1)	82.0 ± 1.0 (d, 1)
Indulin AT	—	—	8.0 ± 0.1 (a, 1)	8.0 ± 0.0 (a, 1)	8.0 ± 0.1 (a, 1)
		DMP	9.7 ± 0.2 (b, 1)	10.3 ± 0.3 (b, 1)	10.5 ± 0.2 (b, 1)
		Guaiacol	9.5 ± 0.1 (b, 1)	9.9 ± 0.1 (b, 1)	10.1 ± 0.2 (b, 1)
	Purified *Cc*Lcc5	—	10.7 ± 0.0 (b, 1)	10.4 ± 0.3 (b, 1)	10.0 ± 0.0 (b, 1)
		DMP	10.9 ± 0.4 (b, 1)	15.8 ± 1.0 (c, 2)	14.0 ± 0.1 (c, 2)
		Guaiacol	12.0 ± 0.0 (b, 1)	12.8 ± 0.1 (b, 1)	13.0 ± 0.0 (b, 1)
	*Cc*Lcc5	—	12.7 ± 0.6 (c, 1)	13.5 ± 1.4 (c, 1)	11.0 ± 0.4 (b, 1)
		DMP	20.9 ± 0.4 (d, 2)	25.9 ± 1.0 (e, 2)	24.0 ± 0.4 (d, 2)
		Guaiacol	16.0 ± 0.2 (c, 1)	16.8 ± 0.2 (c, 1)	17.0 ± 0.0 (c, 1)
	Novozym 51003	—	16.7 ± 0.7 (c, 1)	19.5 ± 2.6 (d, 1)	15.0 ± 1.3 (c, 2)
		DMP	22.9 ± 0.6 (d, 1)	27.9 ± 1.6 (f, 2)	25.0 ± 0.3 (e, 2)
		Guaiacol	18.0 ± 0.4 (c, 1)	18.2 ± 0.3 (c, 1)	18.0 ± 0.5 (c, 1)

a Different letters behind the means ± SD (*n* = 3 samples) represent significant differences (*p* > 0.05) in MWs, compared to the original sizes of lignosulfonate and Indulin AT. Different numbers behind the means ± SD (*n* = 3 samples) represent significant differences (*p* > 0.05) in MWs of samples over the incubation time.

The least effects in this study were visible with the purified basi-laccase *Cc*Lcc5, with an MW increase for lignosulfonate to about 60 kDa and for Indulin AT to about 10 kDa. Addition of either one of the mediators further increased MWs, to about 68 and 16 kDa in the case of DMP and to about 63 and 13 kDa in the case of guaiacol (Table 1). Purified *Cc*Lcc5 was apparently active on both technical lignins in their polymerization, with both mediators improving the reactions on the lignins.

Treatments with purified basi-laccase *Cc*Lcc5 were inferior to treatments with unpurified *Cc*Lcc5 and treatments with Novozym 51003 with the asco-laccase *Mt*L (Table 1). MW values rose with unpurified *Cc*Lcc5 by another 5–6 kDa for lignosulfonate and by up to 3 kDa for Indulin AT. Addition of DMP and of guaiacol boosted the MW further by >20 and 6–9 kDa for lignosulfonate, respectively. The extra increments in case of Indulin AT were about 7–13 and 3–4 kDa. As with purified *Cc*Lcc5, DMP was more effective as a mediator than guaiacol. The spent fungal medium, in which *Cc*Lcc5 was supplied, apparently had an additional effect on better lignin polymerization, as compared to all reactions with purified *Cc*Lcc5. At present, we do not know what was responsible in the medium for the effect, whether it was favorable metabolites produced by the fungus or leftover or converted medium compounds.

Even better results in the polymerization were achieved with commercial Novozym 51003 (Table 1), applied as supplied in the formulation by the company and commonly used in fiberboard production (e.g., Kudanga et al., 2008; Widsten et al., 2009; Euring et al., 2011; Gouveia et al., 2018; Garajová et al., 2021). The MWs of lignosulfonate increased with Novozym 51003 by around 10 kDa as compared to the initial value and with DMP by a further 25–30 kDa and with guaiacol by a further 14–15 kDa. The MW on Indulin AT increased with Novozym 51003 by 8–11 kDa and by a further 6–10 kDa under addition of DMP. Guaiacol had less effect with a maximum of 3 kDa increase in MW. Thus, DMP was also the better mediator with the commercial asco-laccase *Mt*L preparation. The MWs obtained here with Novozym 51003 and lignosulfonate were comparable to those in our former work using the same experimental conditions but with 200-fold higher enzyme concentration and with 10 mM caffeic acid or 10 mM vanillyl alcohol as mediators (Euring et al., 2016a). In LLMS with lignosulfonate, the mediator DMP with the best MW of 97.9 ± 1.8 kDa (Table 1) performed slightly better as in a previous study using caffeic acid with the best MW of 94.3 ± 1.2 kDa (Euring et al., 2016a). Third in performance was guaiacol with the best MW of 83.2 ± 1.2 (Table 1), followed by vanillyl alcohol with the best MW of 80.5 ± 1.1 kDa (Euring et al., 2016a). We are not aware of the exact formulation of the Novozym 51003 brown liquid, but it likely contains spent fungal growth medium as well; the producer refers to potential fermentation smells of preparations and batch-dependent color variations of the liquid (Novozymes, 2007). If, for example, it is not based on extra added stabilizers, additional compounds from the medium may therefore be present, which are helpful for the laccase reactions in the radicalization and polymerization of lignin. However, heat inactivation of Novozym 51003 has shown before that reactions in fiberboard production depended on an active enzyme (Euring et al., 2011), and Sephadex G-25 purified Novozym 51003 was active on *Eucalyptus* kraft lignin in oxidative polymerization (Gouveia et al., 2018). In summary, it is unclear whether the better performance of Novozym 51003 in this experimental setup with technical lignins compared to unpurified basi-laccase *Cc*Lcc5 could be related to parameters provided by the commercial formulation of the *Mt*L enzyme and not directly to the asco-laccase itself.

Time played a role for the results obtained (Table 1). MWs of lignins (lignosulfonate and Indulin AT) were tested after 15, 30, and 60 min of incubation. Without mediators, the differences in increased MW at different incubation times with enzymes, if any, were small and usually non-significant. Only Novozym 51003 showed a slightly higher polymerization of both types of lignins by incubation for 30 min. The MWs were about 70 kDa in the case of lignosulfonate, as in our former study (Euring et al., 2016a), and about 20 kDa in the case of Indulin AT (Table 1). Mayr et al. (2021) similarly reported a 30 min incubation time for Novozym 51003 incubated with lignosulfonate liquor for the best increase in MW, with a reduction in MW in subsequent incubation. In this study, also when the better mediator DMP was added to either the lignosulfonate or the Indulin AT in laccase solutions, a 30 min incubation time was required and also sufficient for the highest polymerization by all enzyme preparations, purified basi-laccase *Cc*Lcc5, unpurified *Cc*Lcc5, and asco-laccase *Mt*L in Novozym 51003. After 60 min incubation in our experiments, there were usually small but significant reductions in MWs (Table 1). Similar tendencies were observed in the former work with other mediators, when 30 min incubation of lignosulfonate with Novozym 51003 in the presence of caffeic acid or vanillyl alcohol resulted in slightly higher MWs than the longer incubation time of 60 min (Euring et al., 2016a). Decreases in MWs of the lignins during the longer incubation time with the laccases probably indicated that lignin depolymerization reactions were then in progress. We interpret the entire situation in such a way that an equilibrium between polymerization and depolymerization of the lignin will be established in the reaction mixtures. Both DMP and guaiacol were applied in this study at 10 mM, with respect to 0.5 g lignin at a relative concentration that is sufficiently low to ensure that the two chemicals acted as mediators in their mode of operation (Huber et al., 2016). Our chromatograms did not reveal significant changes in the MWs of mediators (not further shown), suggesting negligible self- and co-polymerizations with lignin. In contrast to other mediators so far tested, guaiacol in this study showed only nonsignificant differences in MWs of lignosulfonate and of Indulin AT over the incubation time with any of the enzyme preparations applied (Table 1).

**FIGURE 2:**
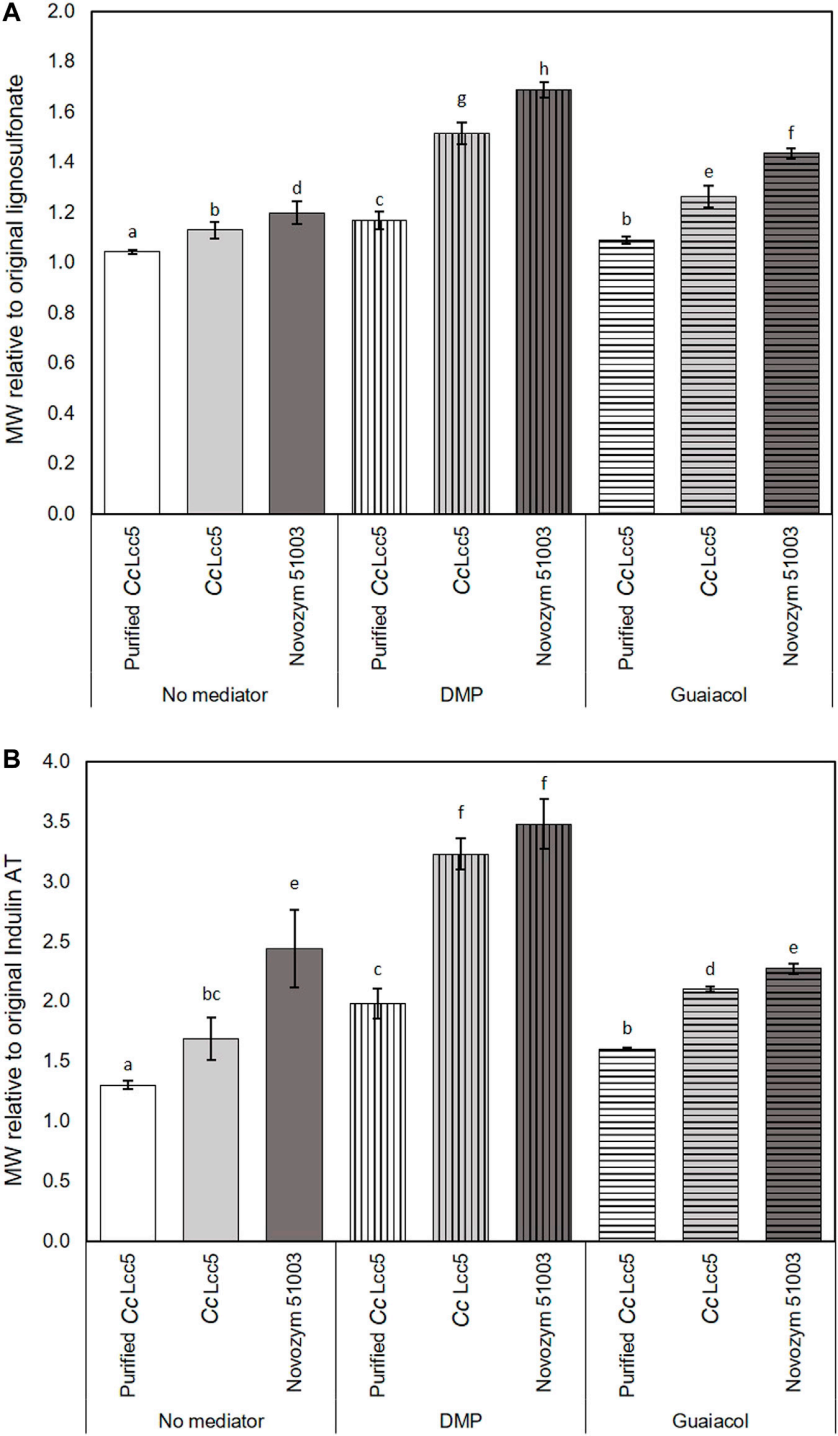
Relative increments in MWs of technical lignins, **(A)** lignosulfonate and **(B)** Indulin AT, by 30 min incubation with different laccase preparations, purified and unpurified *Cc*Lcc5 and Novozym 51003, applied in samples without mediator and in LLMS with either DMP or guaiacol, as calculated from data presented in Table 1. Different letters above bars ± SD (*n* = 3 samples) represent significant differences (*p* > 0.05) in relative increase of MWs compared to the original MW of lignosulfonate and Indulin AT.

The summary in Figure 2 reinforces the high potential of laccase-mediated oxidation of industrial lignins with the best mediator DMP. The figure presents the fold increases in MWs at the optimal 30 min incubation of laccases with DMP acting on lignosulfonate (Figure 2A) and Indulin AT (Figure 2B) as compared to the original sizes of the lignins. Unpurified basi-laccase *Cc*Lcc5 with the mediator DMP showed 1.5- and 3.2-fold increments in the MWs of lignosulfonate and Indulin AT, which were significantly higher than for the purified *Cc*Lcc5 with 1.2- and 2.0-fold increments, respectively. Novozym 51003 with asco-laccase *Mt*L showed a 1.7-fold increment in the MW of lignosulfonate and a 3.5-fold increment in the MW of Indulin AT with the mediator DMP. Absolute increments in MW were thus always much higher with the hydrophilic lignosulfonate (Table 1), but with relative increases for the hydrophobic Indulin AT (Figure 2). These differences link to a fivefold lower absolute size of the original Indulin AT (Table 1) and likely to the different water-based solubilities of the two technical lignins (Lund and Ragauskas, 2001; Goldmann et al., 2019).

### Oxygen Consumption Measurements

**FIGURE 3:**
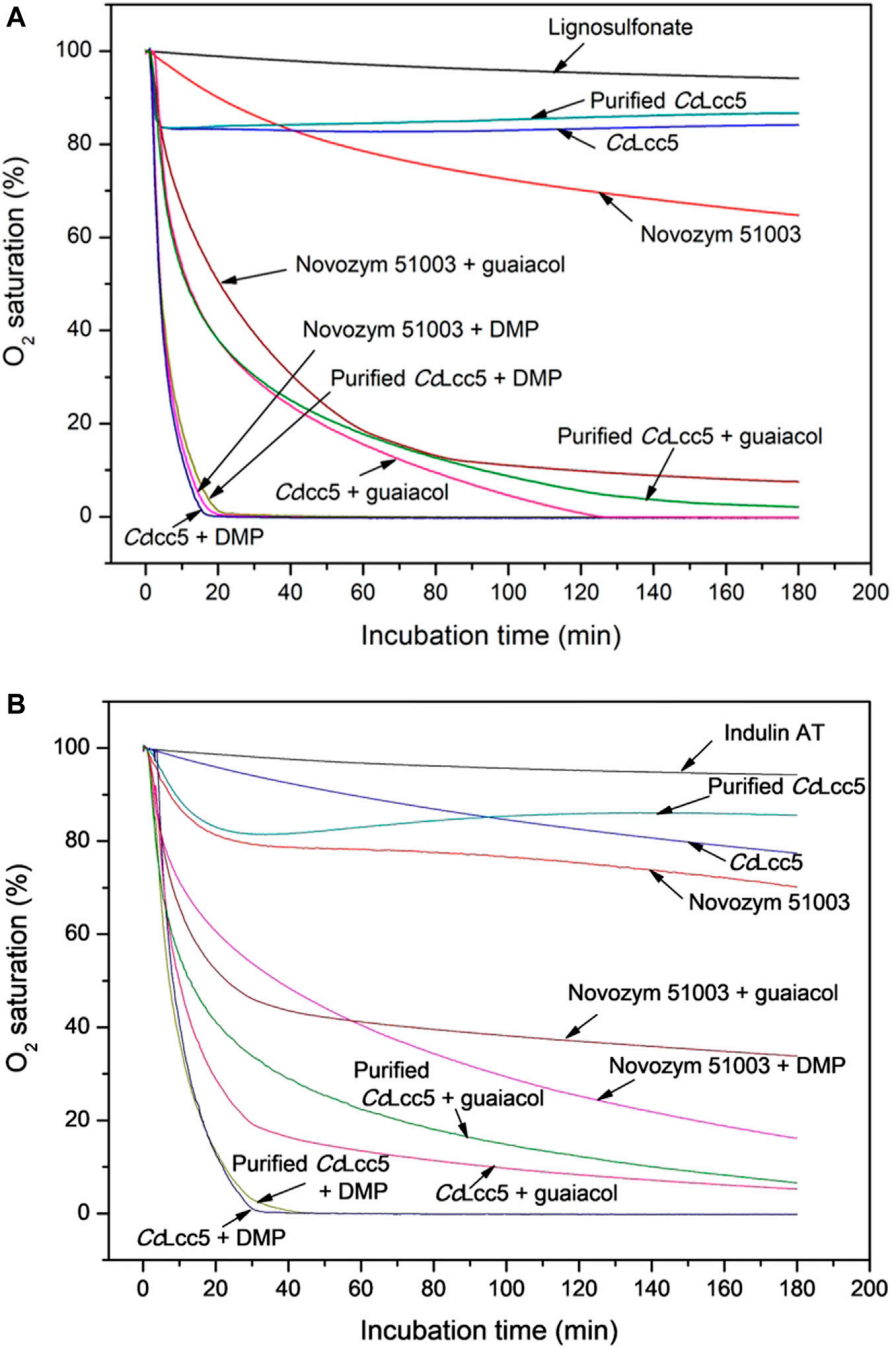
Oxygen consumption curves in samples of technical lignins, **(A)** lignosulfonate and **(B)** Indulin AT, incubated without enzyme or with different laccase preparations, purified and unpurified *Cc*Lcc5 and Novozym 51003, in the absence or, in LLMS, in the presence of a mediator, either DMP or guaiacol. The curves are calculated as average values of three repetitions.

Mediators guaiacol and DMP greatly enhanced the oxidation of lignosulfonate and Indulin AT in LLMS with either of the three laccase preparations, as judged by the increases in lignin MWs (Table 1). This was further analyzed by oxygen consumption tests over 3 h time of incubation in O_2_-saturated McIlvaine buffer (pH 6.0) (Figure 3). Control measurements of pure McIlvaine buffer (pH 6.0) and of technical lignins with only mediators, only mediators, or only enzymes revealed only slight decreases in the oxygen saturation of the buffer, which is about 5%–10% or less at the end of the incubation time (not further shown here for simplicity of figures), similar to that for the technical lignins alone (Figures 3A,B) and to that reported before for the Novozym 51003 formulation (Euring et al., 2016a). Purified or unpurified basi-laccase *Cc*Lcc5 with mediators lead to a maximum of 10% oxygen consumption and Novozym 51003 with the asco-laccase *Mt*L with mediators to a maximum of 25% (not further shown in the figures).

Addition of a laccase (purified or unpurified *Cc*Lcc5 or Novozym 51003) to a technical lignin enhanced the oxygen consumption in all samples (Figures 3A,B), in line with the fact that laccases need O_2_ for their biochemical reactions on their phenolic substrates (Kües and Rühl, 2011; Kües, 2015). Oxygen consumption is a more direct measurement of the biochemical activity of the enzymes on the available substrate than the determination of lignin MWs, which are influenced by possible secondary reactions by mediators, lignin, and other potential compounds in the laccase preparations. The increases in oxygen consumption in lignin–enzyme samples therefore suggested that the enzymes likely acted directly on the technical lignins, as deduced before in other studies for Novozym 51003 and either lignosulfonate (Johansson et al., 2014; Ortner et al., 2015; Euring et al., 2016a; Huber et al., 2016) or kraft lignin (Johansson et al., 2014; Ortner et al., 2015). In this study, enzymatic oxidation of lignosulfonate or Indulin AT by laccases alone showed a maximum of about 30% consumption of the saturated oxygen in the buffer by Novozym 51003 with asco-laccase *Mt*L over the incubation time. Unpurified basi-laccase *Cc*Lcc5 and purified *Cc*Lcc5 in comparison were less consumptive in the presence of a lignin, with between 15% and 20% consumption of the original O_2_ (Figures 3A,B).

The addition of the mediator DMP greatly accelerated and increased the oxygen consumption of all laccase preparations in the presence of lignosulfonate (Figure 3A). Within 20 min of incubation, the whole oxygen in the buffer was consumed with only slight differences, firstly with *Cc*Lcc5 + DMP, followed by Novozym 51003 + DMP and then purified *Cc*Lcc5 + DMP. Enzymatic reactions with DMP proceeded thus with similar efficiency, despite the low overall sequence conservation and dissimilar substrate pocket sequences between the basi- and asco-laccase (Figure 1). Oxygen consumption did not simply add up from the consumption of a laccase with lignosulfonate and the consumption of the same laccase with the mediator. This can be explained by recycling reactions of the oxidized mediators on the lignin, which are then disposed again for repeated oxidation by the enzymes and which is then reflected in the further increased oxygen consumption (Bourbonnais and Paice, 1992; Kües, 2015; Huber et al., 2016).

From tests with a *Trametes hirsuta* basi-laccase with different lignin model compounds and variable mediators (guaiacol, syringaldehyde, erol, veratrol, and adlerol) (Rittstieg et al., 2002) and from oxygen consumption tests with asco-laccase *Mt*L in Novozym 51003, different mediators [acetovanillone, ethylvanillin, ferulic acid, guaiacol, and 4-hydroxybenzoic acid (HBA)], and wood fibers (Kirsch et al., 2015), guaiacol was concluded before to be a good mediator with a high oxidation potential on lignins (both technical and native). Addition of guaiacol in this study to lignosulfonate also increased the oxygen consumption of all the laccase preparations in a similar manner, but with much longer incubation time for full oxygen consumption (approximately 120 min) as compared to the better DMP and a slightly faster reaction time for *Cc*Lcc5 (Figure 3A). The relatively similar oxygen consumption curves between the three laccase preparations with lignosulfonate in presence of guaiacol (Figure 3A), however, indicated similar, albeit less strong, oxidation strengths of the distantly related enzymes for guaiacol as well.

Differences were then seen between the laccase preparations upon addition of mediators to Indulin AT samples (Figure 3B). Considering purified and unpurified basi-laccase *Cc*Lcc5, the mediator DMP had similar effects for both in the oxidation of Indulin AT (Figure 3B) as before in the oxidation of lignosulfonate (Figure 3A). After an incubation time of about 30 min, oxygen was completely consumed in the Indulin AT plus DMP samples (Figure 3B). In the case of mediator guaiacol, too, the oxygen consumption curves were relatively comparable for the purified and unpurified *Cc*Lcc5 (Figure 3B), while they were somewhat delayed in time in comparison to the oxygen consumption curves in the presence of the hydrophilic lignosulfonate (Figure 3A). Furthermore, the two distinct curves for Novozym 51003 oxygen consumption in the presence of Indulin AT and a mediator grouped also closer together (Figure 3B). However, speed and level of oxygen consumption by asco-laccase *Mt*L in presence of Indulin AT and mediators were decreased. These curves of the asco-laccase *Mt*L declined less sharply as compared to those of the different basi-laccase *Cc*Lcc5 applications to Indulin AT with mediators (Figure 3B) and also to those of Novozym 51003 with the lignosulfonate and mediators (Figure 3A). After 180 min of incubation, oxygen consumption leveled out at only 60% with guaiacol and 80% with DMP in the Indulin AT samples with the asco-laccase *Mt*L in Novozym 51003 (Figure 3B).

For interpretation of the results, it is important to note in these experiments that purified and unpurified *Cc*Lcc5 behaved basically the same in all oxygen consumption tests with a lignin and a mediator, regardless of which technical lignin and which mediator were applied (Figures 3A,B). The highly increased oxygen consumption in the presence of a lignin and a mediator suggests that the enzyme catalyzed mainly the oxidation of the added and recycling mediator in the enzymatic reaction, without any impeding effect from the culture medium in the case of unpurified enzyme. Oxygen consumption was influenced for all laccase preparations by the technical lignin applied with a mediator. Generally, lignosulfonate samples reacted faster and stronger in oxygen consumption than samples with Indulin AT (Figures 3A,B). Bibliographic data report a total amount of phenolic hydroxyl groups of 1.57 mmol/g for Ca-lignosulfonate (Jablonský et al., 2015) and a higher amount of 2.49 mmol/g for Indulin AT (Gärtner et al., 1999). A higher content of phenolic hydroxyl groups might seem preferable for catalytic reactions with laccases, with oxidized mediators, and within and between lignin molecules themselves. However, the more accessible aromatic groups in the hydrophilic lignosulfonate with better solubility capacity as compared to the hydrophobic less-soluble Indulin AT could be the reason for the superior reactions encountered with lignosulfonate despite the less-phenolic hydroxyl groups in this technical lignin with larger MW (Johansson et al., 2014; Ortner et al., 2015). We assume that the fundamentally higher potential of Indulin AT due to more phenolic hydroxyl groups is not fully exploited under our test conditions. A complete exploitation would require a suitable chemical pretreatment of the Indulin AT (Karthäuser et al., 2021).

The oxygen consumption of the asco-laccase *Mt*L in Novozym 51003 in the Indulin AT samples with both mediators was significantly negatively affected, compared to that of basi-laccase *Cc*Lcc5 in the Indulin AT samples with the mediators (Figure 3B). Because mediator recycling with unpurified or purified *Cc*Lcc5 in the Indulin AT samples was apparently nearly as good as in the lignosulfonate samples (Figure 3A), it suggests specific inhibitor reactions of Indulin AT to the asco-laccase *Mt*L in Novozym 51003 and its enzymatic functionality. As typical for other asco-laccases and in contrast to basi-laccases, *Mt*L has been seen in crystals to aggregate into dimers via unique interfaces (Ernst et al., 2018) that in part overlap with the sequences of and thus block the phenolic substrate binding pockets required for the mediators to become oxidized (Figure 1). In solution, such enzyme dimerization as a structural feature would thus be unproductive, which is why the active *Mt*L form must be monomeric. Size-exclusion chromatography of *Mt*L (128 kDa) showed that a rapid mono-dimer equilibrium on minute scale may exist in solution (Ernst et al., 2018), but it is unclear whether Indulin AT could promote such blocking dimer formation of *Mt*L. Indulin AT has a high content of low-MW organically bound sulfur as compared to lignosulfonate (Ortner et al., 2015; Hu et al., 2016). The quantitatively most frequent representative of the bound sulfur in Indulin AT is dimethyl-disulfide. This may inhibit the depolymerization of kraft lignin (Daniel et al., 2019). Demethylation of kraft lignin is recommended for activation (Bourbonnais and Paice, 1992; Hu et al., 2011; Venkatesagowda, 2019). In this study, the dimethyl-disulfide in Indulin AT might affect the tested enzyme preparations differentially, resulting then in the lower oxygen consumption of asco-laccase *Mt*L in Novozym 51003 with the recycling mediators. The hydrophobic kraft lignin has a good principal protein binding capacity, likely driven by nonspecific hydrophobic interactions or hydrogen bonding and possibly causing changes in protein conformations (Salas et al., 2013). The unusual outer surface binding of the larger non-phenolic substrate ABTS close to the entrance of the T1-Cu pocket of *Mt*L with largely neutral and hydrophobic residues could indicate particularities of *Mt*L in interactions with larger plant cell wall polymers (Ernst et al., 2018), which may here hinder oxidative enzymatic actions of *Mt*L. Another obvious difference in structure between asco-laccase *Mt*L and basi-laccase *Cc*Lcc5 with possible negative regulatory effects on the enzymatic activity is the C-tail, which *Mt*L has extra (Figure 1). Such C-tails are also typical of asco-laccases and act as a plug to block the water channel of the enzymes (Andberg et al., 2009; Pardo and Camarero, 2015; Ernst et al., 2018; Olmeda et al., 2021). It is conceivable that the function of the plug in asco-laccase *Mt*L can be impaired by irregular structural effects caused by the hydrophilic Indulin AT. This would then provide another explanation for the comparably poor polymerization of Indulin AT observed here with Novozym 51003 (Table 1) and in former studies by others (Ortner et al., 2015; Ortner et al., 2018).

In general, when all results are compared, the speed and final rates of oxygen consumption (Figures 3A,B) correlated not much with the magnitudes of the former results on an increase in MW of technical lignins (Table 1), similar to that observed in other studies (Ortner et al., 2015). At this stage of research, the complexity of all the reactions and interactions that take place in the samples with the enzymes and on the available phenolic natural mediators and with the technical lignins is far too complicated to understand and explain; it is also difficult to reliably predict the quality of outcomes of different LLMS in biotechnological applications.

### Physical–Technological Properties of LLMS-Bonded MDF

The so far described analyses showed that both the commercial Novozym 51003 with asco-laccase *Mt*L and the basi-laccase *Cc*Lcc5 were able to very quickly and effectively polymerize lignosulfonate or Indulin AT as technical lignins in the presence of DMP. DMP (syringol) is a natural phenolic mediator with two *O*-linked methoxyl groups, related to sinapyl alcohol as one phenolic component in lignins. Sterically, it should therefore be more easily accessible by laccases than certain mediators such as HBA, which has only a *p*-carboxyl group, and vanillyl alcohol which has a *p*- and a *m*-substituted carboxyl group (Huber et al., 2016), which is why we considered reducing the amount of enzyme in further application as compared to former LMS- and LLMS-bonded MDF studies with HBA and vanillyl alcohol (Euring et al., 2016a; Kirsch et al., 2016). Based on the results of the GPC and the oxygen consumption measurements, MDF production with LLMS was therefore carried out on pilot plant scale, using either lignosulfonate or Indulin AT, DMP as the natural mediator, no uneconomical enzyme purification, and either unpurified basi-laccase *Cc*Lcc5 or asco-laccase *Mt*L in Novozym 51003 as an enzymatic catalyst, applied at a 200-fold reduced enzyme level (5 kU enzyme per kg atro wood fibers). IB, MOR, and TS of boards were tested to judge the quality of the obtained products (Table 2).

**TABLE 2:** Physical–mechanical properties for 8-mm-thick MDF and a density of 780 kg/m^3^ bonded with laccases, LMS, and LLMS.

Variant			IB^a^	MOR^a^	TS^a^
Glue/enzyme	Mediator	Lignin	(N/mm^2^)	(N/mm^2^)	(%)
—	—	—	0.07 ± 0.01 (a)	7 ± 1 (a)	107 ± 5 (a)
	DMP	—	0.09 ± 0.02 (a)	8 ± 1 (a)	102 ± 5 (a)
	—	Lignosulfonate	0.18 ± 0.02 (b)	11 ± 3 (b)	89 ± 7 (a)
	—	Indulin AT	0.16 ± 0.01 (b)	12 ± 2 (b)	78 ± 3 (b)
	DMP	Lignosulfonate	0.18 ± 0.03 (b)	12 ± 1 (b)	94 ± 3 (a)
	DMP	Indulin AT	0.17 ± 0.02 (b)	12 ± 1 (b)	87 ± 2 (a)
*Cc*Lcc5	—	—	0.12 ± 0.01 (b)	10 ± 2 (b)	84 ± 5 (b)
	DMP	− (LMS)	0.18 ± 0.04 (b)	13 ± 1 (b)	48 ± 5 (d)
	DMP	Lignosulfonate (LLMS)	0.55 ± 0.03 (e)	19 ± 1 (d)	38 ± 2 (d)
	DMP	Indulin AT (LLMS)	0.43 ± 0.01 (d)	17 ± 1 (c)	31 ± 1 (e)
Novozym 51003	—	—	0.21 ± 0.03 (c)	11 ± 2 (b)	56 ± 4 (c)
	DMP	− (LMS)	0.23 ± 0.02 (c)	15 ± 1 (c)	45 ± 4 (d)
	DMP	Lignosulfonate (LLMS)	0.59 ± 0.02 (e)	21 ± 2 (d)	36 ± 1 (d)
	DMP	Indulin AT (LLMS)	0.51 ± 0.02 (e)	17 ± 0 (c)	34 ± 2 (d)
UF K350 10%	—	—	0.75 ± 0.10 (f)	29 ± 2 (e)	19 ± 1 (f)
^a^Required for 8-mm-thick MDF according to DIN EN 622-5 (2010)			≥0.65	≥23	≤17

a Different letters behind the means ± SD represent significant differences (*p* > 0.05) in IB (*n* = 36 per board), MOR (*n* = 18 per board), or TS (*n* = 36 per board) compared to the reference values of MDF, made with neither enzyme nor mediator or technical lignin or conventional binder.

Reference boards pressed only of wood fibers or of fibers with only the mediator DMP were very brittle and had unacceptable physical technological properties, as expected. Adding of technical lignins (lignosulfonate or Indulin AT) with or without DMP significantly improved the consistency and technical quality of boards, which indicates that the lignin during hot-pressing (200°C) plasticizes above the glass transition point and that it glues through polymerization reactions during down-cooling (Felby et al., 1997; Widsten and Kandelbauer, 2008a; Widsten et al., 2009). However, the tested physical–technological parameters still remained below required standard specifications (Table 2). Similar or slightly improved board qualities were achieved under addition of either enzyme, without or, in LMS, with DMP (Table 2). Application of the LMS compared to solely laccases in this study improved TS and MOR (Table 2), comparable to what was previously reported in LMS-MDF production for Novozym 51003 and mediator HBA (Euring et al., 2011; Kirsch et al., 2016; Kirsch et al., 2017).

Treatments of fibers in LLMS with all three components, an enzyme, DMP, and a technical lignin, improved the situation drastically as compared to the treatments with only a laccase and as compared to the LMS performed without extra added lignin. The physical–technological performances of MDF from LLMS came close to the required norms (Table 2). The LLMS boards made with lignosulfonate were equal to or slightly better than those produced with the hydrophobic Indulin AT, except the TS based on the more hydrophilic character of lignosulfonate (Melro et al., 2021). The LLMS boards made with asco-laccase *Mt*L in Novozym 51003 and Indulin AT were somewhat better than those made with basi-laccase *Cc*Lcc5. However, the slightly better values obtained for LLMS boards made with Novozym 51003 and lignosulfonate were nonsignificantly different to those of LLMS boards made with *Cc*Lcc5 and lignosulfonate (Table 2). Overall, the results with LLMS and Novozym 51003 correspond to the better degrees of polymerization in the GPC test with Novozym 51003 as compared to *Cc*Lcc5 (Table 1). It underlines that prominent individual parameters, such as those mentioned here, for example, the excellent oxygen consumption of basi-laccase *Cc*Lcc5 in the presence of Indulin AT compared to the only moderate results by Novozym 51003 (Figure 3B), cannot necessarily predict the ultimately better behavior of an LLMS in a biotechnological application such as in LLMS-MDF production (Table 2).

In summary, boards made with either one of the enzymes, 10 mM DMP, and 10% lignosulfonate were comparatively best (Table 2). IB reached 0.55 N/mm^2^ with basi-laccase *Cc*Lcc5 and 0.59 N/mm^2^ with Novozym 51003, close to the ≥0.65 N/mm^2^ aimed by the standard for 8-mm-thick MDF (DIN EN 622-5, 2010). At the same time, the achieved MOR was highest at 19 N/mm^2^ for *Cc*Lcc5 and at 21 N/mm^2^ for Novozym 51003, compared to the required ≥23 N/mm^2^ for 8-mm-thick MDF (DIN EN 622-5, 2010). The TS was 38% for the LLMS boards made with basi-laccase *Cc*Lcc5 and was 36% for those made with asco-laccase *Mt*L in Novozym 51003, twice as much as envisaged in the standard (≤17%; DIN EN 622-5, 2010), due to the hydrophilic character of the lignosulfonate (Melro et al., 2021). An MDF-specific hydrophobing agent (Sasol HydroWax 138) was added at 1% in this study to face the problem. Future work may increase the concentration up to 2% as it is sometimes used with synthetic binders in the production of special water-resistant MDF (Dunky and Niemz, 2002), while the influence on the adhesiveness of LLMS must be investigated. Felby et al. (2002) previously applied Novozym 51003 on wood fibers with 1% of a wax emulsion. They found the laccase activity in radical formation unaltered by the wax but observed a reduction in mechanical–technological board properties due to wax coating on the fibers that in consequence reduced their adhesion. Work by Kirsch et al. (2015) with an LMS and 2% wax emulsion led to a better TS close to the required norm but lowered IB and MOR compared to the use of 1% wax.

## Conclusion

In this study, basi-laccase *Cc*Lcc5 was compared with the distantly related asco-laccase *Mt*L (31% sequence identity) as a novel enzyme in LLMS-bonded MDF production. Despite their poor sequence conservation and in particular the very different substrate binding domains (Figure 1), these two medium-redox-activity enzymes showed a similarly high potential for use with selected mediators and technical lignins in the wood-based panel industry. Unpurified *Cc*Lcc5 and *Mt*L in the Novozym 51003 formulation performed equally well with 10% lignosulfonate, 10 mM mediator DMP, and only 5 kU enzyme applied per kilogram of atro wood fiber in MDF production on a pilot plant scale. Resulting boards with physical–technological performances came close to standard specifications, indicating the principal usefulness of both enzymes for LLMS-MDF production with the easier-to-process hydrophilic lignosulfonate. The values obtained in LLMS with the massively available hydrophobic Indulin AT were slightly inferior but show a potential for use as a material in a green MDF binder system with either enzyme and the natural mediator DMP. In LLMS with Indulin AT and DMP, basi-laccase *Cc*Lcc5 performance was lower in IB compared to asco-laccase *Mt*L in Novozym 51003 while it was equal in MOR and even better in TS. The reason for these differences remained unclear due to the complexity of potential reactions that may occur in the systems. Determination of lignin MWs via GPC and the oxygen consumption measurements provided variably superior results for *Cc*Lcc5 or Novozym 51003, which do not explain all observations made with the enzymes in the LLMS. For example in the oxygen consumption tests with lignosulfonate, both enzymes were comparably good with the mediator DMP and reacted similarly but with lower efficiency with guajacol. The activity of asco-laccase *Mt*L with both mediators in contrast was negatively affected by Indulin AT. In GPC, in contrast, results on the MW increase of technical lignins were better throughout with Novozym 51003 than with either purified or unpurified basi-laccase *Cc*Lcc5. In these experiments testing the oxidative performances of the distantly related enzymes, guaiacol was always the less effective mediator compared to DMP, which is why it was not further considered in LLMS-MDF production.

In summary, the MDF production using the LLMS systems showed promising results for both kinds of laccases and both types of technical lignins tested but still has space for further optimization, especially concerning the TS of produced boards. Further work on LLMS-MDF production should concentrate on the enzymes for technical improvement and process upscaling, especially on basi-laccase *Cc*Lcc5 and probably also in combination with other natural mediators. Unpurified *Cc*Lcc5 was used here as harvested from fungal cultures, while stabilized industrial enzyme formulations similar to, e.g., Novozym 51003 have to be implemented in order to provide an economical enzyme with good catalytic and technical specifications in large quantities.

## Data Availability

The original contributions presented in the study are included in the article, further inquiries can be directed to the corresponding authors.
